# Actinobacteria from Termite Mounds Show Antiviral Activity against Bovine Viral Diarrhea Virus, a Surrogate Model for Hepatitis C Virus

**DOI:** 10.1155/2015/745754

**Published:** 2015-10-22

**Authors:** Marina Aiello Padilla, Rodney Alexandre Ferreira Rodrigues, Juliana Cristina Santiago Bastos, Matheus Cavalheiro Martini, Ana Caroline de Souza Barnabé, Luciana Konecny Kohn, Ana Paula Trovatti Uetanabaro, Getúlio Freitas Bomfim, Rafael Sanches Afonso, Fabiana Fantinatti-Garboggini, Clarice Weis Arns

**Affiliations:** ^1^Laboratory of Virology, Department of Genetics and Evolution and Bioagents, Institute of Biology, University of Campinas (UNICAMP), P.O. Box 6109, 13083-970 Campinas, SP, Brazil; ^2^Multidisciplinary Center for Chemical Biological and Agricultural Research, University of Campinas (UNICAMP), Avenida Alexandre Cazelatto 999, Betel, 13148-218 Paulínia, SP, Brazil; ^3^University of Santa Cruz (UESC), Campus Soane Nazaré de Andrade, Rodovia Jorge Amado, km 16, Salobrinho, 45662-900 Ilhéus, BA, Brazil; ^4^University of Feira de Santana (UEFS), Avenida Transnordestina, s/n, Novo Horizonte, 44036-900 Feira de Santana, BA, Brazil

## Abstract

Extracts from termite-associated bacteria were evaluated for *in vitro* antiviral activity against bovine viral diarrhea virus (BVDV). Two bacterial strains were identified as active, with percentages of inhibition (IP) equal to 98%. Both strains were subjected to functional analysis via the addition of virus and extract at different time points in cell culture; the results showed that they were effective as posttreatments. Moreover, we performed MTT colorimetric assays to identify the CC_50_, IC_50_, and SI values of these strains, and strain CDPA27 was considered the most promising. In parallel, the isolates were identified as *Streptomyces* through 16S rRNA gene sequencing analysis. Specifically, CDPA27 was identified as *S. chartreusis*. The CDPA27 extract was fractionated on a C18-E SPE cartridge, and the fractions were reevaluated. A 100% methanol fraction was identified to contain the compound(s) responsible for antiviral activity, which had an SI of 262.41. GC-MS analysis showed that this activity was likely associated with the compound(s) that had a peak retention time of 5 min. Taken together, the results of the present study provide new information for antiviral research using natural sources, demonstrate the antiviral potential of *Streptomyces chartreusis* compounds isolated from termite mounds against BVDV, and lay the foundation for further studies on the treatment of HCV infection.

## 1. Introduction

Infections with bovine viral diarrhea virus (BVDV) lead to significant economic losses for cattle producers worldwide. This virus is associated with transient fever and diarrhea as well as disorders that affect milk production [1]. BVDV is a member of the Flaviviridae family and* Pestivirus* genus [2]. While there are differences among the members of this genus, BVDV shares similarities in replication cycle, biology, and genetic organization with hepatitis C (HCV), a member of the same family as BVDV but from the* Hepacivirus* genus [3].

Based on these similarities, BVDV is widely used as a surrogate model for* in vitro* antiviral studies against HCV, as HCV does not efficiently replicate in cell culture [4].

There are no available vaccines for the prevention of HCV infection, and current therapy involves a combination of interferon-R (PEG-INF) and ribavirin (1-*β*-D-ribofuranosyl-1,2,4 triazole-3-carboxamide), a nucleoside analog [5, 6]. In addition to its costliness, this treatment is only effective for HCV genotype 1-infected patients and produces numerous side effects, including psychiatric disorders, hemolytic anemia, and changes in blood cell counts, leading to discontinuation [5, 7]. Protease inhibitors (PI), including boceprevir and telaprevir, have revolutionized the treatment of HCV genotype 1 infections; however, combination treatments using PIs have been associated with adverse events, which also lead to the early termination of therapy [8].

Thus, the development of a low-cost therapy with few collateral side effects is a priority.

Natural products comprise substances with diverse chemical structures, providing a substantial pool of potential new drugs [9, 10].

In traditional medicine, termites and termite mounds have been used to treat different diseases worldwide. The termite is commonly used in northern Brazil and in indigenous communities in different parts of the world, such as in the Pareci and Kadiwéu communities, particularly for the treatment of asthma and dermatological problems [11–13]. Additionally, the use of tea-crushed insects or inhalation of incinerated termite soil has previously been reported in cases of bronchitis, wounds, colds, flu, rheumatism, and other conditions [12]. Recently, the termite has also been described as a source of natural products with antibiotic and antifungal activities [14].

Because of the significant difficulties in treating and preventing HCV and the therapeutic potential of natural compounds with antiviral activities, we analyzed the effects of bacteria collected from different termite mounds against BVDV.

## 2. Materials and Methods

### 2.1. Bacteria

#### 2.1.1. Isolation and Maintenance of Microorganisms

Samples of termite mounds were collected between October 2008 and February 2009 from different municipalities in Bahia, Brazil, including Morro do Chapéu (11°33′08′′S, 41°09′27′′W), Lafaiete Coutinho (13°38′11′′S, 40°12′43′′W), and Feira de Santana (12°16′23′′S, 38°57′19′′W). The samples were placed in sterilized polyethylene bags and immediately transported to the Laboratório de Pesquisa em Microbiologia (LAPEM) at Universidade de Feira de Santana, Bahia, Brazil. A 10 g sample of termite mound was suspended in 90 mL of sterile water and manually homogenized for 2 min. Aliquots of 100 mL (10^−1^ at 10^−5^) were inoculated onto Petri dishes containing three different media: ISP2A (10.0 g soluble starch, 4.0 g yeast extract, 10.0 g malt extract, 4.0 g dextrose, and 20.0 g agar in 1000 mL distilled water; pH 7.0–7.2); CCA (10 g soluble starch, 0.3 g KNO_3_, 2.0 g NaCl, 2.0 g K_2_PO_4_·3H_2_O, 2.0 g MgSO_4_, 0.02 g CaCo_3_, 0.01 g FeSO_4_, and 20.0 g agar in 1000 mL distilled water; pH 7.0–7.2), and ISP3 (HIMEDIA). The culture media were supplemented with cycloheximide (50 mg mL^−1^) to control fungal growth. All plates were incubated at 30°C, and the appearance of colonies for bacterial isolation was assessed daily. Pure cultures were obtained after serial transfer into the same culture conditions as above for isolation. The isolates were maintained at −80°C in 20% glycerol until further use.

#### 2.1.2. Identification of Bacteria through 16S rRNA Gene Sequencing Analysis

After allowing the bacteria to grow on agar plates, genomic DNA was extracted from the pure cultures according to a protocol published by Van Soolingen et al. [15]. The 16S ribosomal DNA gene fragments were PCR-amplified using the primers p10f (5′ GAG TTT GAT CCT GGC TCA G 3′) and 1525R (5′ AAG GAG GTG CC 3 WTC CAR 3′), which were homologous to conserved regions of the bacterial 16S ribosomal RNA (rRNA) gene [16]. Fifty-microliter reaction mixtures containing 50–100 ng genomic DNA, 2 U Taq DNA polymerase (Invitrogen), 1x Taq buffer, 1.5 mM MgCl_2_, 0.2 mM dNTP mix (GE Healthcare), and 0.4 *μ*M of each primer were used. The amplification program included 1 cycle at 95°C for 2 min, 30 cycles at 94°C for 1 min, 55°C for 1 min, 72°C for 3 min, and a final extension at 72°C for 3 min using an Eppendorf thermal cycler. PCR amplification of the 16S rRNA gene fragments was confirmed on a 1% agarose gel stained with SYBR Safe (Invitrogen). The 16S rRNA gene fragments were further purified using mini columns (GFX PCR DNA and Gel Band Purification Kit, GE Healthcare) and sequenced using an automated sequencer (ABI3500xL). The sequencing reactions were performed using a BigDye Terminator v3.1 Sequencing Kit (Life Technologies). The primers used in the sequencing reactions were 10f (5′ GAG TTT GAT CCT GGC TCA G 3′), p765f (5′ AGA TAC ATT CCT GGT AG 3′), p782r (5′ ACC AGG GTA TCT AAT CCT GT 3′), 1100R (5′ GGC CTC AGG GTT GTT G 3′), and 1525R (5′ AAG GAG GTG CC 3 WTC CAR 3′). The obtained sequences were processed using the Phred/Phrap/Linux version of the CONSED program for the assembly of contigs [17, 18]. The final obtained sequence was approximately 1100 bp and was compared against sequences in GenBank (http://www.ncbi.nlm.nih.gov/BLAST/) and Ribosomal Data Project II 9.0 (http://rdp.cme.msu.edu/index.jsp). Sequences retrieved from the databases were aligned using the CLUSTAL X program [19], and phylogenetic analyses were conducted using the MEGA program, version 5 [20]. An evolutionary distance matrix was calculated using a previously described model [21], and a phylogenetic tree was constructed based on evolutionary distances using the neighbor-joining method [22], with values calculated from 1000 bootstrap resamples [23].

### 2.2. Extracts

#### 2.2.1. Crude Extracts

A total of 90 bacterial extracts were obtained using liquid-liquid extraction [3]. Briefly, a preinoculum of the isolates was cultured in 7 mL of isolation medium (Nutrient Broth, NB) and incubated at 30°C at 120 rpm. After 48 h, the total volume was transferred to an Erlenmeyer flask containing 50 mL of the same medium and maintained at the same temperature and rotation for 48 h. Subsequently, 50 mL of the bacterial suspension was transferred to a glass jar containing 500 mL of the same medium and maintained under the same conditions as above for four weeks. Then, 500 mL of P.A. grade ethyl acetate was added, and the mixture was triturated in a blender at high-speed rotation. After 24 h, the mixture was filtered using a Buchner funnel containing a PTFE frit-layer of Celite (diatomaceous earth), and the organic phase was recovered in an Erlenmeyer flask using a separator funnel. Cell debris and culture medium were retained in the aqueous phase, and potentially biologically active metabolites were recovered in the organic phase. The organic phase was transferred to a round-bottom flask.

The organic solvent was removed using a Buchi Rotavapor R-215 with vacuum at 37°C until the solvent was completely dry. Subsequently, the extracts were suspended in ethyl acetate and water and transferred to new glass tubes using Pasteur pipettes. These tubes were placed in a Savant vacuum centrifuge (model 210A SpeedVac Plus SC) for evaporation, and the samples were freeze-dried. After complete drying, the extracts were dissolved in 10% dimethylsulfoxide (DMSO) and maintained at −4°C until further use in antiviral activity assays.

#### 2.2.2. Fractionation

The extract presenting the best results, that is, the highest inhibition percentage (IP) and highest selectivity index (SI), was selected for fractionation to identify the potential active compound(s) responsible for its antiviral activity.

The extracts were fractionated using Phenomenex Strata Reversed Phase C18-E SPE cartridges containing 500 mg of adsorbent (sorbent lot number S201-141). Each cartridge was preconditioned with 100% methanol (3 mL) and equilibrated with 100% water (3 mL). Subsequently, each sample was loaded onto the top of the cartridge using a Pasteur pipette at 30 mg/mL sorbent mass, followed by elution with 3 mL of a water/methanol mixture in a gradient starting from the first fraction. The following fractions were sequentially collected: (1) water/methanol (95/05), (2) water/methanol (90/10), (3) water/methanol (80/20), (4) water/methanol (70 : 30), and (5) water/methanol (50 : 50). At the end of the procedure, the cartridge was rinsed with 100% methanol to remove unbound material, which was designated fraction 6. The fractions were stored at 8°C until antiviral activity analysis using GC-MS coupling.

### 2.3. Antiviral Activity Assay

#### 2.3.1. Virus and Cell Lines

The Madin-Darby bovine kidney (MDBK) cell line was used for inoculation with the BVDV strain Cepa Singer. The virus was propagated in monolayer culture using minimal essential medium (MEM) containing Earle's salts. The MDBK cells were supplemented with 10% equine serum (ES). Medium without serum was used when the virus was incubated with the MDBK cells.

#### 2.3.2. MTT Assay

The MTT assay is a sensitive* in vitro* assay for the measurement of cell proliferation or cell viability reduction. To perform the assay, the MDBK cells were cultured in 96-well tissue culture plates. The tetrazolium compound MTT (3-[4,5-dimethylthiazol-2-yl]-2,5-diphenyltetrazolium bromide) was then added to the wells, and the plates were further incubated. MTT is reduced to insoluble purple formazan crystals in metabolically active cells. An aliquot of 150 *μ*L DMSO was subsequently added to each well to solubilize the crystals, and the absorbance was read at 540 nm using a spectrophotometer [24, 25]. The data were analyzed after plotting cell number versus absorbance to quantitate changes in cell proliferation. The rate of tetrazolium reduction is proportional to the rate of cell proliferation and indirectly indicates reductions in cell viability in response to virus infection.

#### 2.3.3. Titration of the Virus

Cells were seeded onto 96-well culture plates at a density of 10^5^ cells/mL and subsequently incubated at 37°C for 24 h in a humidified atmosphere containing CO_2_. Tenfold serial dilutions of the virus stocks were prepared in culture medium, and the cells were infected with the diluted virus. After additional incubation (1-2 days), the cytopathic effect was recorded. The 50% tissue-culture infective dose (TCID_50_) per mL was calculated as previously described [26].

#### 2.3.4. Antiviral Activity

Antiviral activity was determined based on inhibition of the cytopathic effect. All experiments were performed in quadruplicate. Briefly, to evaluate inhibition, cells were seeded onto 96-well culture plates. After incubation for 24 h, the medium was replaced with 100 *μ*L of MEM(E) containing 50 *μ*g/mL bacterial extract, and 50 *μ*L of 100 TCID_50_/50 *μ*L of virus was added and incubated for 72 h. The controls comprised untreated infected cells (virus at 100 TCID_50_/50 *μ*L), treated noninfected cells (extract control), and untreated noninfected cells. The cytopathic effect was observed, and the extracts possessing antiviral activity were identified. Antiviral activity was determined using an MTT assay to quantify the protection conferred by a cell extract against the virus. The protection percentage was calculated using the following formula [27]: (absorbance of treated cells − absorbance of the control virus)/(absorbance of the cellular control − absorbance of the control virus) × 100. The antiviral activity was initially evaluated with a single dose of 50 *μ*g/mL against 100 TCID_50_/mL of virus. Extracts with activities higher than 90% were considered promising. However, only extracts with IPs higher than 98% were considered active and selected for further examination.

To confirm antiviral activity, a concentration-response curve using different concentrations of extract in the presence of 100 TCID_50_/mL of virus was constructed using the MTT assay to determine the 50% inhibitory concentration (IC_50_). The IC_50_ was calculated from the concentration-response curve using linear regression analysis. The results were obtained from triplicate assays using at least five extract concentrations. The 50% cytotoxicity concentration (CC_50_) was calculated as [(*A* − *B*)/*A*] × 100, where *A* and *B* represent the OD_540_ values of untreated and treated cells, respectively. The percentage protection was calculated as [(*A* − *B*) × 100/(*C* − *B*)], where *A*, *B*, and *C* indicate the absorbance values of the extract, virus, and control cells, respectively. Each IC_50_ value was defined as the effective concentration necessary to reduce the absorbance of infected cells by 50% compared with the cell and virus controls. The CC_50_ and IC_50_ values represented the averages of three assays using five concentrations within the inhibitory ranges of the compounds. The therapeutic index (i.e., SI) was defined as CC_50_/IC_50_.

#### 2.3.5. Viral Infection Cycle Staging

Cells and viruses were incubated with active extracts at different stages during the viral infection cycle to determine the antiviral mechanism. The cells were pretreated with bacterial samples prior to viral infection (pretreatment); viruses were incubated with the bacterial samples prior to infecting the cells (virucidal action); or the cells were infected and incubated together with the virus before the addition of the bacterial samples (posttreatment). The samples were used at the maximum noncytotoxic concentration. The antiviral activity was expressed as the titer (TCID_50_/*μ*L) and IP, as previously described by Koseki et al. [28]. The IP was calculated using the formula (IP) = (1 − *T*/*C*) × 100, where *T* is the antilog of the extract-treated viral titers and *C* is the antilog of the control (without extract) viral titers. An IP greater than or equal to 97% was considered positive.

### 2.4. GC-MS Analysis

The crude extracts and active fractions were analyzed using GC-MS. The analyses were performed on an Agilent 6890N gas chromatograph (Palo Alto, CA, USA) interfaced with an Agilent 5975 Mass Selective Detector and an HP 7683B automatic injector, fitted with a fused methyl silicon HP-5MS column (30 m × 0.25 mm i.d., 0.25 *μ*m film thickness) containing 5% phenyl and 95% methylsiloxane. Helium was used as a carrier gas at a flow rate of 1.0 mL/min. The oven temperature was maintained at 150°C for 2 min and subsequently increased to 240°C at a rate of 5°C/min, followed by an increase to 300°C at a rate of 10°C/min. It was then maintained constant for at least 34 min. The injector temperature was maintained at 280°C, and the split ratio was adjusted to 1 : 50. Mass spectral (MS) data were obtained under the following conditions: ionization potential 70 eV, 50–480 amu scan range, 2 sec scan time, detector temperature 300°C, and solvent delay 3 min. The samples were solubilized in ethyl acetate at a concentration of 20 mg/mL and injected into a gas chromatograph column. The constituents of the extracts were identified by comparing the mass spectral data and retention times with corresponding data obtained from the literature and the NIST 2005 computerized MS databank, using 90% as the minimum identification criterion.

### 2.5. Statistical Analysis

The results are expressed as the means ± SEM. Significantly different effects of the tested extracts on the inhibition of virus replication were compared with the control group using Student's *t*-test, with significance considered at *p* ≤ 0.05.

## 3. Results

A total of 90 extracts from different bacterial species previously collected from termites in three regions of Bahia in northeastern Brazil (Lafaiete Coutinho, Feira de Santana, and Morro do Chapéu) were examined. The culture medium used for bacterial growth (NB medium, Oxoid) was used as a control to ensure that there was no interference with the tested compounds produced from the bacteria.

**Figure 1:**
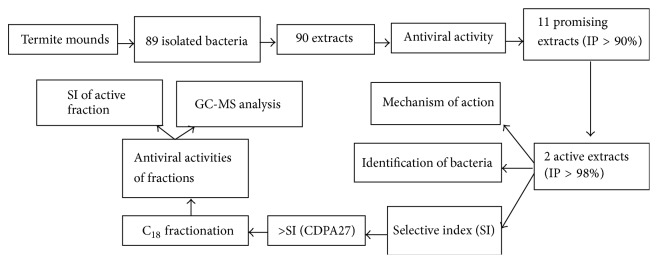
Experimental design employed in this study.

Among the extracts analyzed, 11 samples showed IP values against BVDV that were greater than or equal to 90%. A flowchart of the experiments is shown in Figure 1.

**Figure 2:**
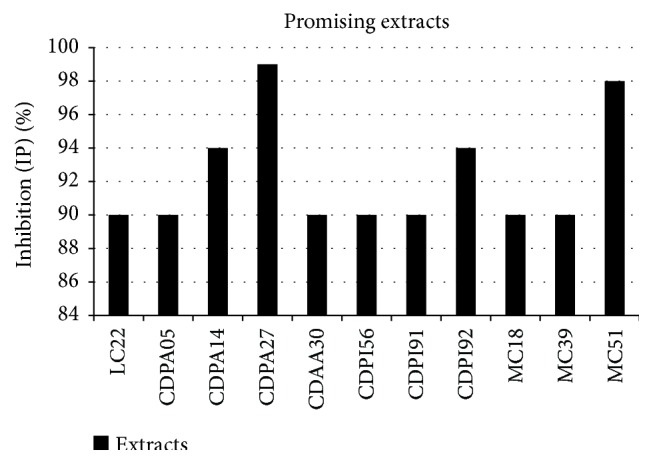
Percentages of inhibition (IP) of the promising extracts.

The extracts listed in Figure 2 showed promising results. The active extracts with IP values greater than 98% included CDPA27 and MC51. The stage of the viral replication cycle during which the extracts exerted their actions was identified, and the SI values (i.e., CC_50_/IC_50_) of the compounds, which indicate their potential to inhibit viral replication without inducing cell damage, were calculated. Thus, a direct relationship could be established between SI value and the potential of an extract as an antiviral compound. To identify the stage of the viral replication cycle during which an active compound operated, three different treatments were administered: (1) the cells were infected with virus for 1 h, followed by the addition of extract to evaluate the viral replication phase (posttreatment); (2) the virus was pretreated with extract prior to infecting the cells (virus inactivation); and (3) extract was added to the cells prior to viral infection to evaluate viral adsorption effects (pretreatment). The SI results and identified mechanisms of action are detailed in Table 1.

**Figure 3:**
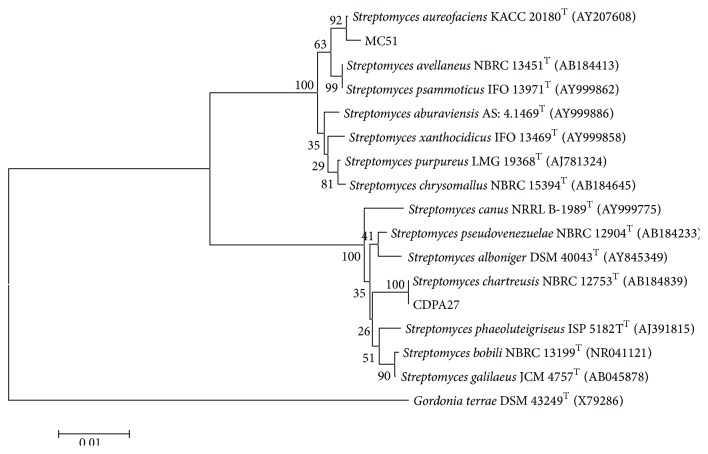
Phylogenetic tree based on the analyses of 16S ribosomal RNA gene sequences of Actinobacteria (Kimura 2-parameter model, neighbor-joining algorithm with 1000 bootstrap resamples). The sequence of the 16S ribosomal RNA gene from* Gordonia terraeT* (X79286) was used as an outgroup.

In addition, we conducted a phylogenetic analysis of the active bacterial compounds based on the ribosomal operon to identify these microorganisms. This technique is useful for the identification of many bacterial species. In the present study, the strain with the highest degree of activity against BVDV, strain CPDA27, was identified as* Streptomyces chartreusis*. Strain MC51 was also identified as a member of the* Streptomyces* genus; however, it was not possible to identify the species of this strain. The phylogenetic tree that was constructed using the above data is presented in Figure 3.

**Figure 4:**
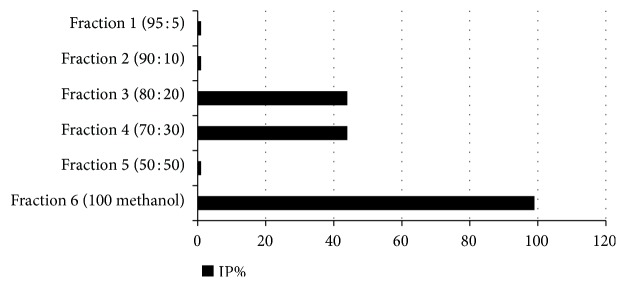
Percentage of inhibition (IP), selectivity index (SI), concentration inhibiting 50% cell growth (CC_50_), and concentration inhibiting 50% viral replication (IC_50_) of the active fraction (see Table 2).

According to the above results, the* S. chartreusis* extract had the best IP and SI values. Thus, this strain was selected for further analysis via fractionation using a C18-E cartridge, and the fractions were reevaluated. The results are shown in Figure 4 and Table 2. Analyzing the IP values of the fractions revealed that fraction 6 (100% methanol) contained the compounds responsible for the observed antiviral activity (Figure 4). The SI of the active fraction, highlighted in Table 2, was 262.41, supporting the use of this sample in future assays.

**Figure 5:**
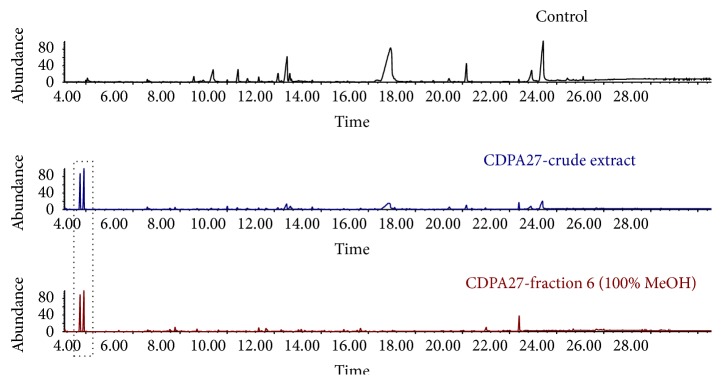
Chromatographic analyses of the control, CDPA27 (crude extract), and fraction 6 (100% MeOH).

To identify the compounds responsible for the antiviral activity described in the present study, the crude bacterial extract, the active fraction, and a control sample of the culture medium without bacteria were analyzed using GC-MS. It was possible to identify the compounds present in both the crude extract and the active fraction and those that were not present in the control sample, suggesting that these compounds were solely derived from* S. chartreusis* fermentation (Figure 5).

Compared with the control, peaks were observed at certain retention times that corresponded to the compounds derived exclusively from bacterial fermentation and that were potentially responsible for antiviral activity. The chromatographic analysis of the CDPA27 extract revealed that the compounds produced by the bacteria appeared at the beginning of the chromatogram. These same compounds were also identified in active fraction 6 (100% methanol).

Thus, these results suggest that the antiviral activity against BVDV described in the present study is associated with the compounds that produced peaks with 5 min retention times.

Although the compounds identified in the present study were thoroughly analyzed, it was not possible to detect similarities between the compounds represented by each peak on the mass fragmentogram and the compounds present in the utilized databases.

## 4. Discussion

Approximately 3% of the world's population (170 million people) is afflicted with chronic HCV infections, which pose a risk for the development of cirrhosis, hepatocellular carcinoma, and liver failure [29–31].

The search for new therapeutic agents to control virus infection is one of the highest priorities in virological research [32], and an increasing number of recent studies have focused on utilizing natural sources to solve medical challenges [33].

Despite the popular use of termites and termite mounds in populations from different regions of the world [12], the antifungal [34] and antibiotic properties [35] of these treatments have only recently been examined. In the present study, we examined the potential antiviral activities of termite-associated bacteria.

A major component of the microbiota of several species of termites is Actinobacteria, which are present in the intestines and on the body surfaces of these insects [36, 37]. Actinobacteria, also known as actinomycetes, are Gram-positive bacteria that produce a variety of secondary metabolites considered to be promising candidates for active natural compounds [35].

In the extracts tested in the current study, the strains CDPA27 and MC51 were the most promising, and phylogenetic analysis identified both strains as* Streptomyces*. This genus has also consistently been described to produce secondary metabolites, such as those obtained from* Streptomyces* sp.* K15*, with significant antimicrobial activities against* Botrytis cinerea* [38]. Additionally, other* Streptomyces* species, such as* S. mutabilis*, which was isolated from soil samples in Saudi Arabia, exhibit promising antituberculous activity [39], and Raveh et al. [40] reported the antiviral activity of a compound isolated from marine sediments obtained from* S. kaviengensis* against western equine encephalitis virus that inhibits the mitochondrial electron transport chain.

The most promising extract in the current study was derived from the fermentation of* S. chartreusis*. Leach et al. [41] first described this species as being responsible for the production of chartreusin, a compound with antibiotic properties against many Gram-positive species, such as* Bacillus subtilis* and* Brucella bronchiseptica*, and Gram-negative species, such as* Salmonella typhosa*,* Escherichia coli,* and* Proteus vulgaris*; chartreusin has also shown activity against mycobacteria. In 1958, Miyakawa et al. [42] evaluated the antiviral activities of several antibiotics against influenza A (strain RP_8_) and further confirmed the significant activity of this compound. Moreover, chartreusin has also been described to possess antitumor activity against murine L1210 and P388 leukemias and B16 melanoma [43]. Furthermore, Aoyama et al. [44] described a minor component of crude chartreusin (3-demethylchartreusin) as a new antitumor antibiotic.

In the present study, the results of antiviral activity assays revealed that CDPA27 affects viral replication; that is, it acts as a posttreatment. This result is consistent with the antitumoral mechanism of chartreusin, which binds to DNA and inhibits the activity of topoisomerase II, interfering with subsequent stages of replication [45]. However, GC-MS analysis did not detect overlaps between database results and the compounds of interest that were present in the fractions and crude extract of this bacterial strain. Chartreusin is described in the utilized database, thereby eliminating the possibility that the antiviral activity described in the present study can be attributed to this compound (Figure 4, Table 2).

In conclusion, the data obtained in the present study were used to identify a bacterial species isolated from termites, that is, a promising producer of secondary metabolites with antiviral activities against BVDV, which serves as a surrogate model of HCV. From this study, it can be concluded that Actinomycetes collected and isolated from termite mounds can be used as sources of pharmacologically active compounds that may be used for the synthesis of new drugs to treat patients with hepatitis C. However, further studies are needed to identify the active compound(s) in the extracts that were evaluated in this study.

## Figures and Tables

**Table 1:** Potential mechanism, selectivity index (SI), 50% cytotoxicity concentration (CC_50_), and concentration inhibiting 50% viral replication (IC_50_) of the active extracts.

Virus	Extract	CC_50_	IC_50_	SI	Mechanism of action
BVDV	CDPA27	409.3	139.8	3.50	Posttreatment
	MC51	32.31	16.99	1.90	Posttreatment

BVDV: bovine viral diarrhea virus; CC_50_: concentration inhibiting 50% cell growth; IC_50_: concentration inhibiting 50% viral replication.

**Table 2:** 

Active fraction	CC_50_	IC_50_	SI
Fraction 6 (100% MeOH)	227.8	0.8681	262.4121

CC_50_: concentration inhibiting 50% cell growth; IC_50_: concentration inhibiting 50% viral replication; SI: selectivity index.
